# Beyond osteopoikilosis: a blended skeletal dysplasia associated with dual *LEMD3* and *ANO5* variants

**DOI:** 10.1210/jcemcr/luag286

**Published:** 2026-10-07

**Authors:** Arun Mariappan, Vivek Jha, Amal Shaharyar, Aravind Sekar, Harpreet Singh, Sanjay Kumar Bhadada

**Affiliations:** Department of Endocrinology, Postgraduate Institute of Medical Education and Research (PGIMER), Chandigarh 160012, India; Department of Endocrinology, Postgraduate Institute of Medical Education and Research (PGIMER), Chandigarh 160012, India; Department of Endocrinology, Postgraduate Institute of Medical Education and Research (PGIMER), Chandigarh 160012, India; Department of Pathology, Postgraduate Institute of Medical Education and Research (PGIMER), Chandigarh 160012, India; Department of Nuclear Medicine, Postgraduate Institute of Medical Education and Research (PGIMER), Chandigarh 160012, India; Department of Endocrinology, Postgraduate Institute of Medical Education and Research (PGIMER), Chandigarh 160012, India

**Keywords:** osteopoikilosis, gnathodiaphyseal dysplasia, LEMD3, ANO5, blended skeletal dysplasia, pathological fracture

## Abstract

Osteopoikilosis is a rare autosomal dominant sclerosing bone dysplasia caused by pathogenic variants in *LEMD3*, which is typically asymptomatic and incidentally detected on radiographs. Gnathodiaphyseal dysplasia is an extremely rare skeletal disorder characterized by diaphyseal sclerosis, bone fragility, and pathological fractures. The coexistence of 2 genetically distinct skeletal dysplasias in a single individual is exceptionally uncommon. A 15-year-old female presented with progressive gait disturbance and a right hip intertrochanteric fragility fracture. Radiological evaluation demonstrated widespread mixed sclerotic and geographic lytic lesions with predominant involvement of the appendicular skeleton. Serum 25-hydroxyvitamin D was reduced with secondary hyperparathyroidism, whereas the calcium–phosphate profile was normal. Bone biopsy revealed a fibro-osseous lesion. Clinical exome sequencing identified a heterozygous pathogenic nonsense variant in *LEMD3*, consistent with osteopoikilosis, and a heterozygous likely pathogenic missense variant in *ANO5*, associated with gnathodiaphyseal dysplasia. This case describes a rare blended skeletal phenotype associated with coexisting *LEMD3* and *ANO5* variants, presenting with osteopoikilosis, bone fragility, fibro-osseous histopathology, and mixed lytic–sclerotic skeletal lesions. The phenotype could not be fully explained by either disorder alone, highlighting the complexity of dual molecular diagnoses and the importance of comprehensive genetic evaluation in patients with unexplained skeletal lesions and pathological fractures

## Introduction

Inherited skeletal dysplasias are a heterogeneous group of disorders that affect bone formation, remodeling, and mineralization. These conditions may present during childhood or adolescence with skeletal deformities, abnormal radiological findings, or pathological fractures [1]. Osteopoikilosis is a rare benign sclerosing bone dysplasia with autosomal dominant inheritance and most commonly caused by pathogenic variants in the *LEMD3* gene [2]. Radiographically, they are characterized by multiple small, symmetric sclerotic bone islands that are typically located in the periarticular regions of long bones, pelvis, and tarsal bones [3]. Most individuals with osteopoikilosis are asymptomatic, and it is usually detected incidentally during imaging performed for other reasons.

Gnathodiaphyseal dysplasia is an extremely rare skeletal disorder associated with pathogenic variants in the *ANO5* gene, characterized by diaphyseal sclerosis, cortical thickening, bone fragility, and susceptibility to pathological fractures [4]. With the increasing use of next-generation sequencing, blended phenotypes caused by pathogenic variants in more than 1 gene are being increasingly recognized [5]. Such dual genetic diagnoses may lead to atypical clinical presentations that cannot be explained by either disorder alone [6]. Recognition of these blended phenotypes is important for accurate diagnosis and genetic counseling.

Here, we report an adolescent female with a genetically confirmed dual diagnosis with blended skeletal phenotype of osteopoikilosis and gnathodiaphyseal dysplasia, presenting with pathological fracture and extensive mixed sclerotic and lytic skeletal lesions.

## Case presentation

A 15-year-old female student presented with a 9-month history of progressive difficulty in walking. She also reported intermittent nocturnal bilateral thigh cramps and exertional fatigue for 2 months. An unintentional weight loss of approximately 2 kg over 10 months was noted. There was no history of fractures, bone pain, hearing impairment, dental abnormalities, or recurrent infections. She subsequently sustained a low-energy fall during routine household activity, followed by acute pain in the right hip region without associated swelling or neurovascular compromise.

The patient attained menarche at 13 years of age and with regular menstrual cycles. She was born of a nonconsanguineous marriage. Both parents had hypertension with onset after 40 years of age. Her elder sister was clinically asymptomatic. An older brother had died in infancy due to neurodevelopmental delay. There was no known family history of fractures or diagnosed skeletal disorders.

General examination revealed a congenital hyperpigmented patch over the right upper thigh. Dermoscopic evaluation demonstrated linear and whorled hypermelanosis. Systemic examination was unremarkable. No craniofacial abnormalities, jaw swelling, or dental anomalies were identified.

## Diagnostic assessment

Plain radiography of the pelvis revealed a right intertrochanteric fracture of the proximal femur. Multiple geographic lytic and sclerotic lesions were observed involving the pelvis, bilateral femoral head and neck (Fig. 1). A whole-body skeletal survey demonstrated predominant widespread involvement of the appendicular skeleton than axial skeleton, with mixed sclerotic and lytic lesions affecting the epiphyses, metaphysis, and diaphysis (Fig. 1). Serum calcium was 9.7 mg/dL (2.42 mmol/L; reference range, 8.5-10.5 mg/dL [2.12-2.62 mmol/L]), and serum phosphorus was 3.6 mg/dL (1.16 mmol/L; reference range, 2.5-4.5 mg/dL [0.81-1.45 mmol/L]). Alkaline phosphatase was 236 U/L (3.93 μkat/L; reference range, 44-147 U/L [0.73-2.45 μkat/L]). Serum 25-hydroxyvitamin D was severely reduced at 6.3 ng/mL (15.7 nmol/L; reference range, 20-50 ng/mL [50-125 nmol/L]), with elevated intact parathyroid hormone at 130 pg/mL (13.7 pmol/L; reference range, 10-65 pg/mL [1.6-6.9 pmol/L]), consistent with secondary hyperparathyroidism. Bone turnover markers were markedly elevated, with procollagen type 1 N-terminal propeptide (P1NP) of 436 ng/mL (436 μg/L; reference range, 15-80 ng/mL [15-80 μg/L]) and β-C-terminal telopeptide (β-CTX) of 3147 pg/mL (3147 ng/L; reference range, 100-700 pg/mL [100-700 ng/L]) (Table 1). Hemoglobin was 11.2 g/dL, with normal total leukocyte and platelet counts. Erythrocyte sedimentation rate was 16 mm/h. Thyroid function tests, serum prolactin, and IGF-1 levels were within normal limits. Bone turnover markers were markedly elevated (Table 1).

**Figure 1.**
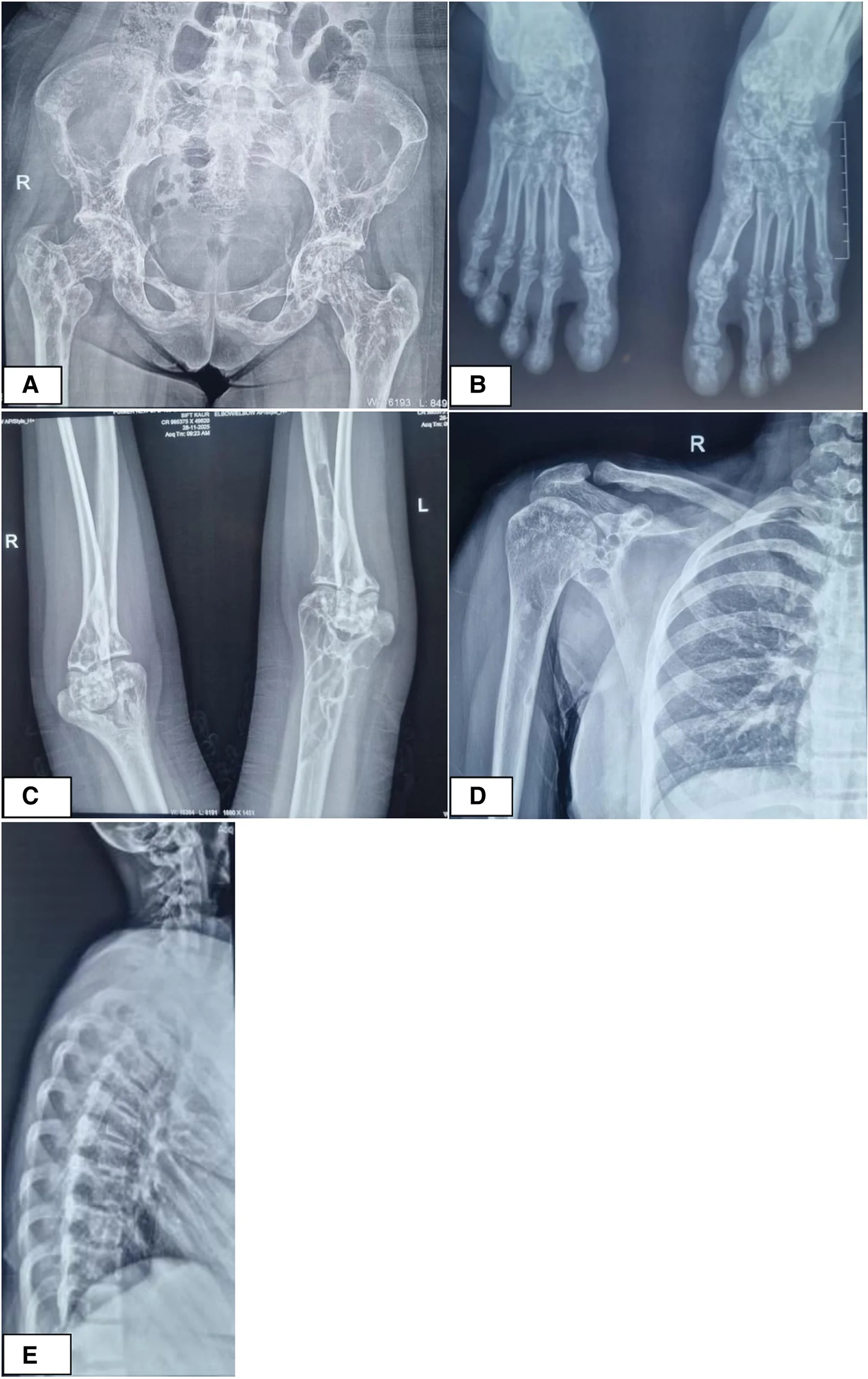
Radiographic findings of the proband. (A) Pelvic radiograph demonstrating multiple sclerotic and lytic lesions involving the iliac bones and proximal femora. (B) Radiograph of the feet showing numerous symmetric sclerotic bone islands involving the tarsal bones and metatarsals. (C) Radiographs of the long bones demonstrating multiple geographic lytic lesions with few sclerotic foci. (D) Radiograph of the humerus revealing scattered sclerotic foci within the proximal and distal metaphyseal regions with few lytic lesions. (E) Radiograph of the lateral spine showing preserved vertebral morphology and alignment without significant abnormalities.

**Table 1:** Biochemical profile of the proband

Parameter	Values (SI/conventional)	Reference range
Serum calcium	9.7 mg/dL (2.42 mmol/L)	8.5-10.5 mg/dL (2.12-2.62 mmol/L)
Serum phosphorus	3.6 mg/dL (1.16 mmol/L)	2.5-4.5 mg/dL (0.81-1.45 mmol/L)
Alkaline phosphatase (ALP)	236 U/L (3.93 µkat/L)	44-147 U/L (0.73-2.45 µkat/L)
25-Hydroxyvitamin D	6.3 ng/mL (15.7 nmol/L)	20-50 ng/mL (50-125 nmol/L)
Intact parathyroid hormone (PTH)	130 pg/mL (13.7 pmol/L)	10-65 pg/mL (1.6-6.9 pmol/L)
Procollagen type 1 N-terminal propeptide (P1NP)	436 ng/mL (µg/L)	15-80 ng/mL (15-80 µg/L)
β-C-terminal Telopeptide (β-CTX)	3147 pg/mL (ng/L)	100-700 pg/mL (100-700 ng/L)

Abbreviations: ALP, alkaline phosphatase; PTH, parathyroid hormone; P1NP, procollagen type 1 N-terminal propeptide; β-CTX, beta-C-terminal telopeptide.

Dual-energy X-ray absorptiometry demonstrated preserved lumbar spine bone mineral density (BMD) with reduced femoral neck and total hip BMD (Table 2). Lumbar spine radiographs were normal with no structural abnormalities that could have affected BMD measurements. Trabecular bone score was 1.456, consistent with preserved trabecular microarchitecture.

**Table 2:** Bone mineral density measurements of the proband

Site	BMD (g/cm^2^)	Z score	Reference range
Lumbar spine (L1-L4)	1.061	+1.0	−2.0 to +2.0
Femoral neck	0.430	−3.8	−2.0 to +2.0
Total hip	0.580	−3.0	−2.0 to +2.0

Abbreviation: BMD, bone mineral density.

Bone marrow examination revealed normocellular marrow with trilineage hematopoiesis and thickened subcortical bony trabeculae.

Whole-body ^18^F-sodium fluoride positron emission tomography/computed tomography (PET/CT) (Fig. 2A) demonstrated multiple metabolically active skeletal lesions involving both axial and appendicular skeleton with predominantly lytic lesions and punctate calcifications. Whole-body ^18^F-fluorodeoxyglucose PET/CT (^18^F-FDG PET/CT) (Fig. 2B) also revealed multiple FDG-avid lytic skeletal lesions correlating with abnormalities seen on conventional imaging.

**Figure 2.**
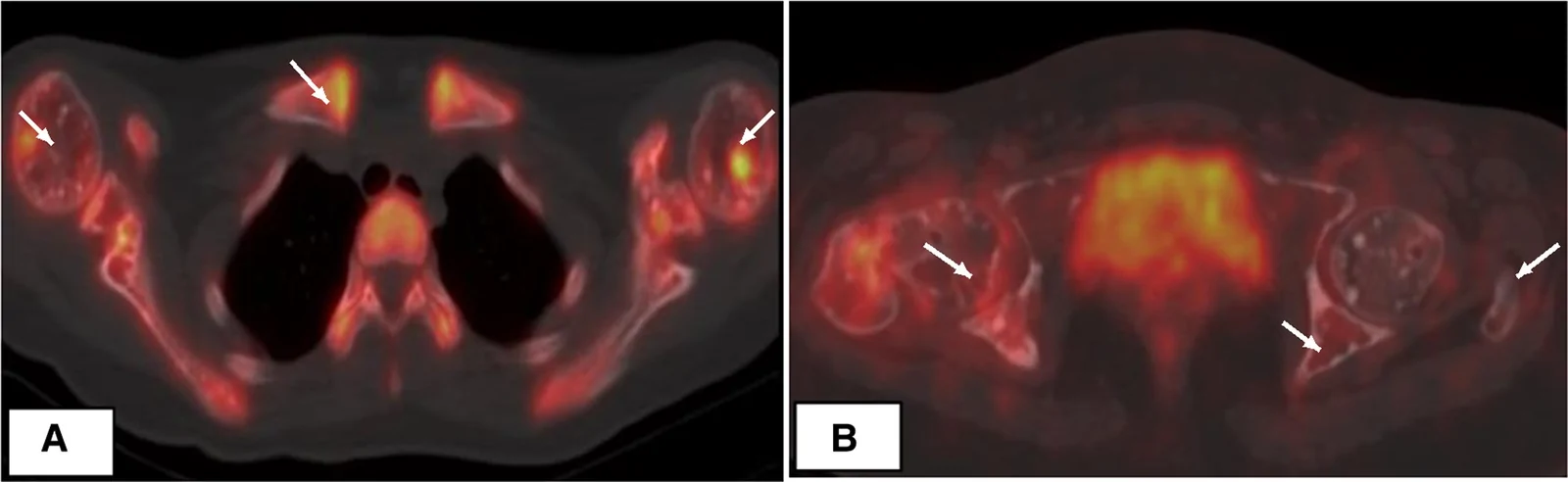
(A) Axial fused ^18^F sodium fluoride positron emission tomography/computed tomography (PET/CT) image at level of shoulder showing increased tracer uptake in bilateral humeral heads, clavicles, and scapulae, suggesting multiple lytic lesions. (B) Axial fused ^18^F-fluorodeoxyglucose PET/CT (^18^F-FDG PET/CT) image at the level of pelvis showing increased FDG uptake in pelvic bones with multiple geographic lytic lesions and punctate calcifications.

Histopathological examination of a biopsy obtained from a representative lytic lesion in the right iliac bone showed irregular bony trabeculae within fibrocollagenous stroma without osteoblastic rimming, consistent with a fibro-osseous lesion (Fig. 3).

**Figure 3.**
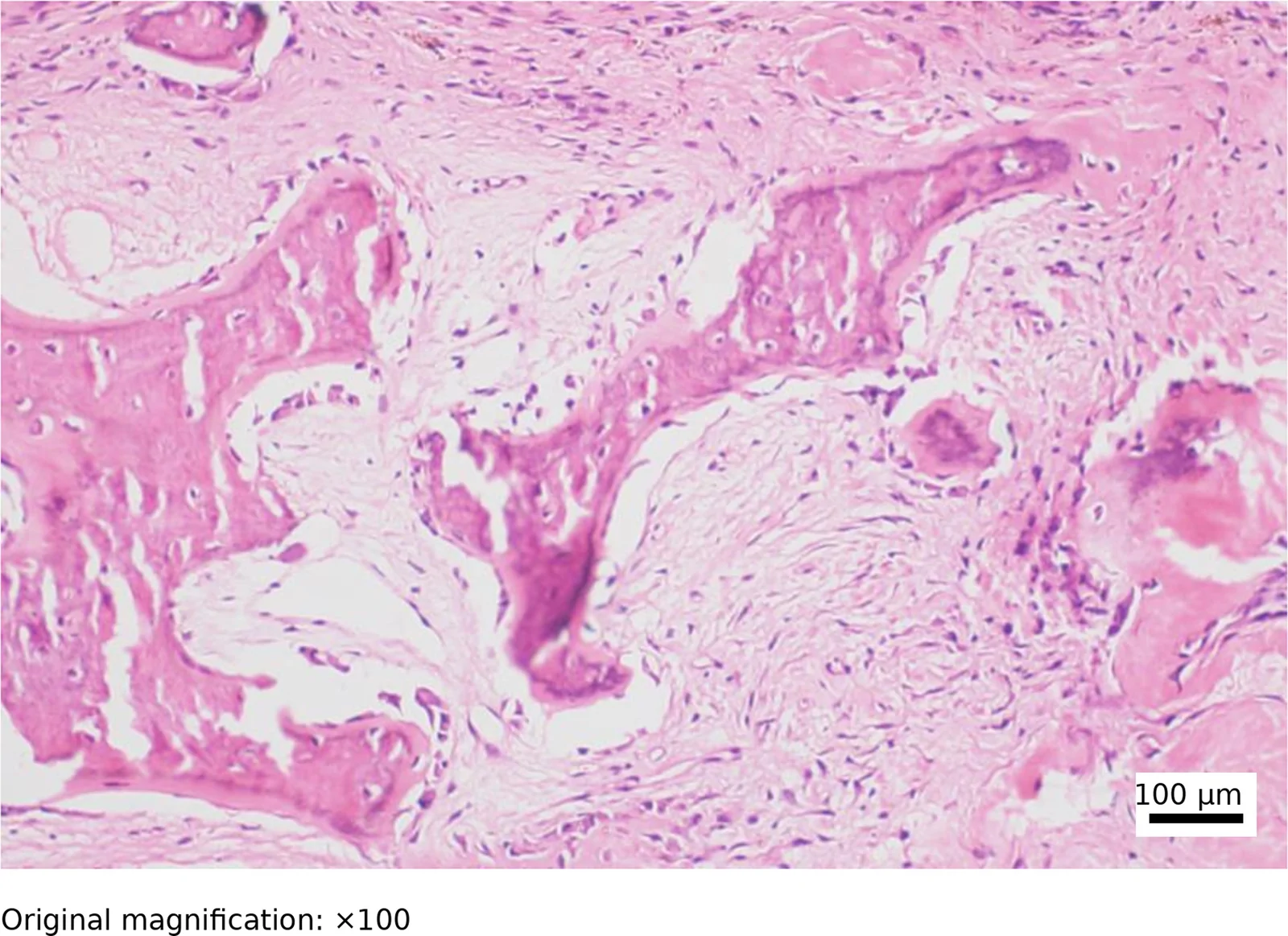
Histopathology of bone biopsy (hematoxylin and eosin stain) showing irregularly thickened bony trabeculae with a fibrocollagenous tissue in the intratrabecular space with no osteoblastic rimming/atypical cells/granuloma, suggestive of fibro osseous lesion. Original magnification, ×100; scale bar, 100 μm.

Clinical exome sequencing revealed a heterozygous pathogenic nonsense variant in *LEMD3* (NM_014319.5: c.1963C>T; p.Arg655Ter; exon 7), consistent with osteopoikilosis. Additionally, a heterozygous likely pathogenic missense variant in *ANO5* (NM_213599.3: c.1823T>C; p.Ile608Thr; exon 17) was detected, reported in association with gnathodiaphyseal dysplasia and classified as likely pathogenic by American College of Medical Genetics and Genomics-based laboratory interpretation. No pathogenic variants in *GNAS* were identified on exome sequencing.

Radiological and genetic evaluation of first-degree relatives was performed. Radiographs of the patient's mother demonstrated multiple well-defined periarticular sclerotic lesions involving the hands, wrists, feet, and pelvis, consistent with osteopoikilosis (Fig. 4). She was clinically asymptomatic with no history of fractures or bone pain, and biochemical evaluation showed normal serum calcium, phosphorus, and alkaline phosphatase levels. Clinical exome sequencing confirmed that she carried the same heterozygous variants in *LEMD3* and *ANO5*. In contrast, the patient's older sister had normal skeletal imaging and did not harbor these variants, supporting maternal inheritance with autosomal dominant transmission.

**Figure 4.**
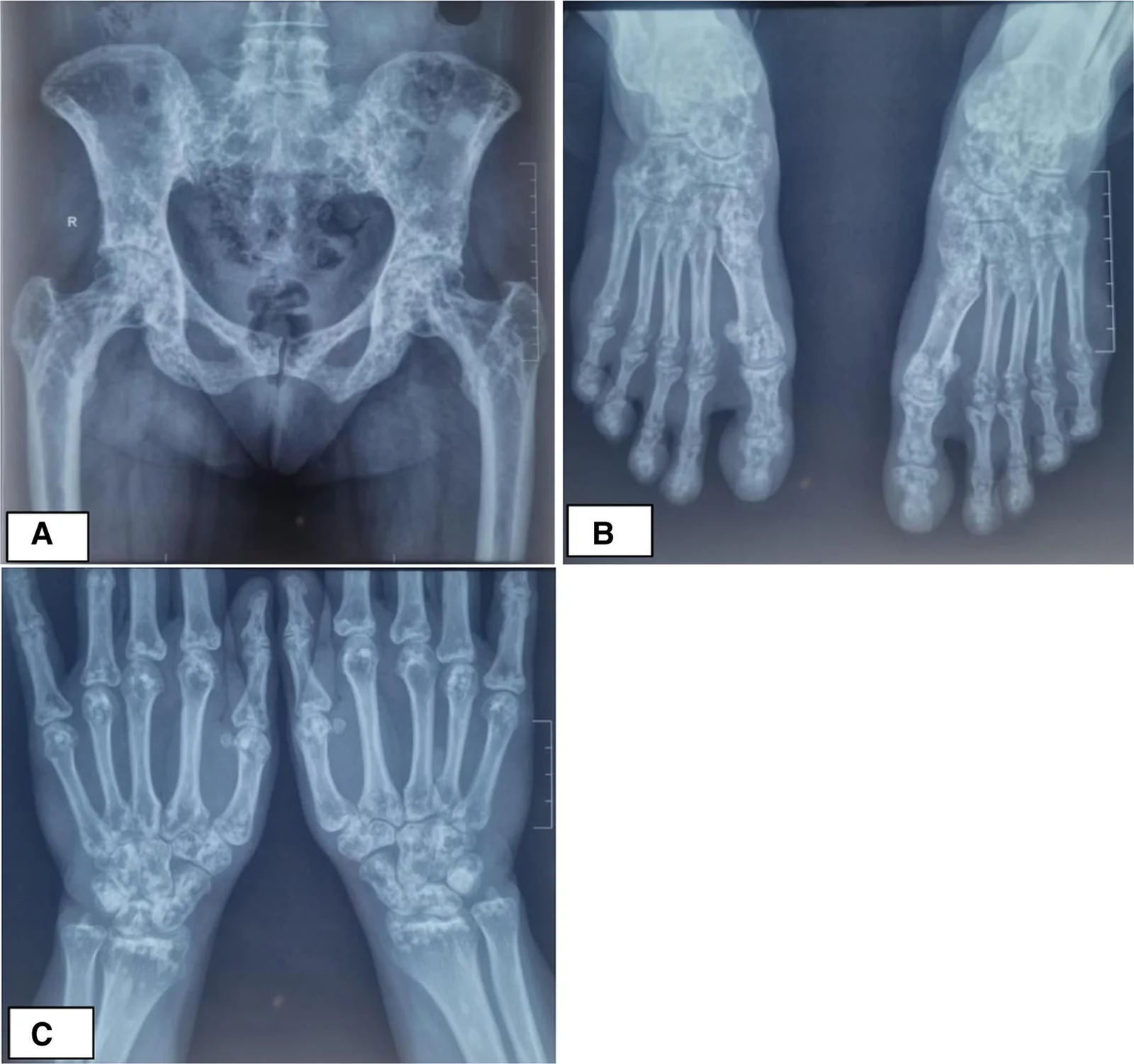
Radiographic findings of mother. (A) Anteroposterior radiograph of the pelvis demonstrating multiple sclerotic foci involving the pelvic bones and proximal femora. (B) Anteroposterior radiograph of both feet showing diffuse sclerotic foci involving the tarsal bones, metatarsals, and phalanges bilaterally. (C) Anteroposterior radiograph of both hands showing diffuse sclerotic foci involving carpal bones, metacarpals, and phalanges bilaterally.

## Treatment

The patient was initially treated with calcium and cholecalciferol supplementation to correct vitamin D deficiency and secondary hyperparathyroidism. Right intertrochanteric hip fracture was managed conservatively with immobilization. Following biochemical improvement and adequate vitamin D repletion, single dose of intravenous zoledronate (5 mg) infused over 30 minutes was administered.

## Outcome and follow-up

At 3-month follow up, the patient showed progressive clinical improvement and was able to ambulate without support. Biochemical evaluation showed improvement in vitamin D deficiency and secondary hyperparathyroidism with decline in bone turnover markers with P1NP decreasing to 279 ng/mL (279 µg/L) and β-CTX to 1471 pg/mL (1471 ng/L). Radiological evaluation demonstrated progressive fracture healing.

## Discussion

Osteopoikilosis is a rare benign sclerosing bone dysplasia characterized by multiple small, well-defined sclerotic bone islands predominantly involving the periarticular regions of the appendicular skeleton, inherited in an autosomal dominant manner and most commonly associated with heterozygous pathogenic variants in the *LEMD3* gene. *LEMD3* encodes an inner nuclear membrane protein that acts as a negative regulator of transforming growth factor-β (TGF-β) and bone morphogenetic protein signaling pathways. Loss of *LEMD3* function leads to dysregulated osteoblastic activity and abnormal deposition of lamellar bone within cancellous bone, resulting in the characteristic enostotic lesions. The lesions are usually stable over time and are most often discovered incidentally during imaging performed for unrelated indications.

In contrast, pathogenic variants in the *ANO5* gene have been implicated in skeletal fragility disorders including gnathodiaphyseal dysplasia [7]. *ANO5* encodes anoctamin-5, a transmembrane protein, involved in membrane repair, intracellular calcium homeostasis, and osteoblast differentiation [8, 9]. Experimental studies suggest that disruption of ANO5 function impairs osteoblast activity and bone remodeling, through altered cellular metabolism and calcium signaling pathways [10], potentially contributing to structural bone weakness and an increased risk of pathological fractures.

Several features of this case are not readily explained by either osteopoikilosis or gnathodiaphyseal dysplasia alone. Extensive multifocal lytic lesions are atypical for both disorders, whereas skeletal fragility is unusual in osteopoikilosis. These observations support a blended skeletal phenotype rather than a classical manifestation of either disorder.

Severe vitamin D deficiency with secondary hyperparathyroidism may also have contributed to skeletal fragility. However, as mineralization parameters were not assessed, a histological diagnosis of concomitant osteomalacia could neither be confirmed nor excluded. Therefore the skeletal phenotype and fracture risk are likely to be multifactorial involving both metabolic bone disease and the underlying skeletal dysplasia.

An additional notable feature in this case was the fibro-osseous lesion identified within lytic iliac bone. Although fibro-osseous lesions of the jaws are considered a hallmark of gnathodiaphyseal dysplasia, phenotypic variability has been increasingly recognized in *ANO5*-related skeletal disease, and longitudinal studies suggest that jaw lesions may be subtle or absent early in disease course and become more apparent with age [11]. In our patient, the presence of a fibro-osseous lesion, skeletal fragility, and *ANO5* variant raises the possibility of ANO5-associated skeletal involvement. However, the absence of characteristic jaw lesions, together with the uncertain pathogenicity of the identified variant, limits the strength of a definitive diagnosis of classical gnathodiaphyseal dysplasia. These findings are therefore more consistent with a complex blended skeletal phenotype associated with coexisting *LEMD3* and ANO-5 variants.

Osteopoikilosis is generally associated with normal bone metabolism and preserved bone strength, whereas data regarding BMD in gnathodiaphyseal dysplasia remain limited. Notably, the patient's mother, who harbored the same genetic variants and exhibited classical osteopoikilosis, had normal BMD and biochemical parameters. Therefore, combination of markedly reduced hip BMD, skeletal fragility, and pathological fracture observed in the proband is unlikely to be explained by either disorder alone and may reflect the blended skeletal phenotype together with coexisting metabolic bone disease.

The striking difference in clinical severity between the proband and her mother highlights the variable expressivity and incomplete penetrance frequently observed in autosomal dominant skeletal dysplasias. Such variability may result from the influence of additional modifier genes, epigenetic factors, environmental influences, or biomechanical loading differences affecting bone remodeling dynamics. The coexistence of variants in 2 genes involved in bone homeostasis may also have contributed to the atypical presentation.

Management was challenging because no established treatment guidelines exist for osteopoikilosis, ANO5-associated skeletal disease or their coexistence. The presence of a fragility fracture, extensive lytic lesions, high bone turnover, and fibro-osseous histopathology supported the use of intravenous zoledronate. This approach was informed by published experience with antiresorptive therapy in fibro-osseous skeletal disorders, including fibrous dysplasia.

Reports describing the coexistence of pathogenic variants in 2 independent skeletal dysplasia genes are rare but are increasingly being recognized in genomic studies of patients with atypical skeletal presentations. Identification of such blended phenotypes is clinically important, as they may explain unusual clinical features or increased disease severity compared with classical single-gene disorders.

Although both *LEMD3* and *ANO5* play important roles in skeletal homeostasis, evidence for a direct biological interaction between these pathways is lacking. Therefore, the unusual phenotype observed in our patient is best interpreted as a blended skeletal presentation arising from the combined effects of 2 distinct genetic defects rather than a demonstrated synergistic molecular mechanism.

This case highlights a rare blended skeletal phenotype associated with coexisting *LEMD3* and *ANO5* variants. The presence of identical variants in the patient's asymptomatic mother, who exhibited classical osteopoikilosis, underscores the intrafamilial phenotypic variability in autosomal dominant skeletal dysplasias. This report illustrates how dual molecular diagnoses can generate atypical phenotypes that are not readily explained by a single genetic disorder and highlights the value of comprehensive genetic evaluation, family screening, and careful assessment of coexisting metabolic bone disease in patients with unexplained skeletal lesions and pathological fractures.

## Learning points

- Coexistence of pathogenic variants in genes regulating bone homeostasis, such as *LEMD3* and *ANO5*, may produce blended skeletal phenotypes with atypical manifestations including pathological fractures.
- Intrafamilial phenotypic variability, with genetically affected but asymptomatic relatives, highlights the role of incomplete penetrance and modifier genetic effects in skeletal dysplasias.
- Comprehensive genetic evaluation and family screening are crucial in patients with unexplained skeletal lesions to ensure accurate diagnosis, appropriate counseling, and long-term surveillance.

## Contributors

A.M., V.J., A.S., A.S., H.S., and S.K.B. were involved in the diagnosis, clinical management of the patient, and preparation and submission of the manuscript. All authors contributed to critical review and approved the final version of the manuscript.

## Data Availability

Data sharing is not applicable to this article as no datasets were generated or analyzed.
